# Mechanism of endogenous hormones regulating gallic acid biosynthesis during the development of buds and leaves in tea plant (*Camellia sinensis*)

**DOI:** 10.3389/fpls.2025.1553266

**Published:** 2025-03-07

**Authors:** Yutao Shi, Xiaofeng Lu, Qingying Song, Huan Sun, Wujing Shen, Ruiqi Huang, Jiapeng Huang, Yanfen Wei, Fumin Xiang, Xi Wang, Yanming Tuo, Jinke Lin, Yunfei Hu

**Affiliations:** ^1^ College of Horticulture, Fujian Agriculture and Forestry University, Fuzhou, China; ^2^ College of Tea and Food Sciences, Wuyi University, Wuyishan, China; ^3^ Anxi College of Tea Science, Fujian Agriculture and Forestry University, Fuzhou, China

**Keywords:** *Camellia sinensis*, buds and leaves, development stage, gallic acid, biosynthesis, plant hormone signal transduction

## Abstract

Gallic acid (GA), as a precursor of Epigallocatechin-3-gallate (EGCG) biosynthesis in tea plant, is one of the important components of tea flavor and has various health benefits. However, the mechanism of endogenous hormones regulating GA biosynthesis during the development of buds and leaves of tea shoots is still unclear. In this study, the buds and leaves of five different developmental stages of tea shoots were used as test materials to explore the mechanism of endogenous hormone signaling pathway regulating GA biosynthesis. The results showed that the decrease of D-erythrosyl-4-phosphate content and the increase of shikimic acid content affected the accumulation of GA content during the development of tea shoots. Jasmonic acid, abscisic acid, auxin, cytokinin, and gibberellin inhibited GA biosynthesis by down-regulating the expression of two *CsaroDEs* through twenty-three plant hormone signal transduction factors, such as *CsMYC2*, *CsSNRK2, CsARR-A*, and *CsDELLA*, respectively, which mediated the downregulation of sixteen transcription factors, such as *CsMYB44*, *CsMYB108*, and *CsC2C2*. *CsMYC2* and *CsSNRK2* co-mediated the downregulation of the expression of *CsMYB44* and *CsMYB108* in response to changes in endogenous JA and ABA content, respectively, and inhibited the expression of *CsaroDE*, thereby co-regulating GA biosynthesis. *CsMYC2* may be a key interworking site for the endogenous Jasmonic acid and abscisic acid signaling pathways to jointly regulate GA biosynthesis. Our findings revealed the potential mechanism of endogenous hormones regulating GA biosynthesis during the development of buds and leaves of tea shoots and provided a scientific basis for the regulation of tea quality.

## 1 Introduction

Tea has become one of the three most popular non-alcoholic beverages in the world because of its unique flavor and good health benefits (Liang et al., 2021; Xing et al., 2024). Gallic acid (GA) is a phenolic acid substance, which is the main component of polyphenols in tea plant. It is an important component that constitutes the flavor of tea and gives tea health benefits (Badhani et al., 2015; Zhang et al., 2023). As a precursor for the biosynthesis of EGCG, which is the core quality and health care component of tea, GA and EGC are catalyzed by serine carboxypeptidases (*CsSCPL*) to form EGCG (Zhao et al., 2022). In addition, GA can also be converted into other metabolites such as methyl gallate in tea plants. Studies have shown that GA and its derivative methyl gallate have strong inhibitory effects on the two main tea plant diseases induced by *Pseudopestalotiopsis camelliae-sinensis* and *Colletotrichum camelliae* and play an important biological function in tea plants (Zhou et al., 2020). The content of GA in tender leaves was higher than that in old leaves, and there were differences in different varieties and different months of tea plants (Shen et al., 2019; Zeng et al., 2020). Subcellular analysis showed that GA mainly accumulated in peroxisomes in tea plant cells. At present, the research on GA mainly focuses on the relationship between GA and tea quality and the health benefits of GA, and there are few reports on the regulation of GA biosynthesis.

Phytohormones are signaling and bioactive molecules produced in higher plants, and the growth and development of higher plants are closely regulated by phytohormones (Zhao et al., 2021). It has been shown that phytohormones have a significant effect on the accumulation of gallic acid content in plants. The addition of TDZ and BA promoted GA accumulation during ginkgo cell suspension culture (Dong and Zhan, 2011); exogenous JA, SA, and ETH treatments significantly increased the accumulation of GA content in Chinese cabbage and affected the expression of GA biosynthesis-related genes (Yadav and Singh, 2023); exogenous melatonin treatment significantly increased GA content in grape berries (Yang et al., 2020); and MEJA treatment alone increased GA content in broccoli by 25.8% (Moreira-Rodríguez et al., 2017); exogenous melatonin treatment significantly increased GA content in rice (Xie et al., 2021); GR24 treatment for 48 h significantly increased GA content in young leaves of tea plant (Yu et al., 2024).

Although the accumulation of GA during the development of tea shoots and the regulation of plant hormones on GA biosynthesis in tea plants have been preliminarily confirmed, the regulation mechanism of endogenous plant hormones on GA biosynthesis during the development of tea shoots is still unclear. In this study, the buds and leaves of tea shoots at different developmental stages were used as the research material. Seven types of endogenous plant hormone components were determined by Liquid chromatography-tandem mass spectrometry (LC-MS/MS), and the relationship between GA content and endogenous hormones in buds and leaves of tea shoots at different developmental stages and their regulatory mechanisms were explored by integrated metabolomics and transcriptomics analyses. In particular, the potential mechanism of co-regulation of GA biosynthesis by jasmonic acid and abscisic acid was clarified. This study aimed to reveal the mechanism of endogenous hormones regulating GA biosynthesis during the development of buds and leaves of tea shoots, and to provide a scientific basis for the improvement of tea quality.

## 2 Materials and methods

### 2.1 Plant materials

The tea plant cultivar ‘*Camellia sinensis* var. Huangdan’, grown in Meizhuang Village, Huqiu Town, Anxi County, Fujian Province (24°54′29′′N, 117°51′42′′E), were used in this study. Samples from five leaf positions including the apical bud (Bud), first leaf (L1), second leaf (L2), third leaf (L3), fourth leaf (L4) of shoots were collected on April 4, 2023. More than 60 individual seedlings were randomly selected from the planting area, and 300~400 g of fresh buds and leaves from different parts of the crown were randomly picked. The fresh leaf samples were evenly divided into two parts after picking. One was immediately frozen in liquid nitrogen for 30 min and stored in a refrigerator at -80 °C for plant hormone content, metabolome detection, and transcriptome sequencing. The other one was microwave-deactivated and dried at 85 °C in a tea dryer. After crushing, it was passed through a 40-mesh sieve and stored in a refrigerator at -20 °C for the detection of gallic acid content. Each group contained three samples.

### 2.2 Determination of GA content

Standard sample of GA (Sigma-Aldrich, USA) was used to make 0.2, 0.4, 0.6, 0.8, and 1.0 mg·mL^-1^ solutions in 70% methanol to construct standard curves. 0.2 g of tea powder was put into a 10 mL centrifuge tube, and 5 mL of 70% methanol solution preheated to 70 °C was added. After mixing, it was immediately moved into a 70 °C water bath, extracted for 10 min, and cooled to room temperature. After centrifugation at 4000 r·min^-1^ for 5 min, the supernatant was moved into a 10 mL volumetric flask. The tea residue was added with 5 mL of 70% methanol solution for the second extraction, and the above steps were repeated. Consolidate the supernatant with 70% methanol to 10 mL, then 1 mL supernatant was taken to another 10 mL volumetric flask and fixed to 10 mL with stable liquid. The solution was filtered with 0.45 μm filter membrane and injected into C18 column (5 μm, 4.6 mm×250 mm, Phenomenex, USA), GA content was determined using high-performance liquid chromatography coupled with ultraviolet-visible spectroscopy (HPLC, Waters Alliance e2695) according to a previous study (Lin et al., 2017).

### 2.3 Quantification of phytohormones

The fresh samples were ground into powder and stored at −80 °C until needed. 50 mg of the plant sample was weighed into a 2 mL plastic test tubes, snap-frozen in liquid nitrogen, and dissolved in 1 mL of methanol/water/formic acid (15: 4: 1, V/V/V). 10 μL of internal standard mixing solution (100 ng·mL^-1^) was added to the extraction solution as an internal standard (IS) for quantification. The mixture was vortexed for 10 min, centrifuged for 5 min, and the supernatant was transferred to a clean plastic microtube, evaporated to dryness and dissolved in 100 μL of 80% methanol (V/V), then passed through a 0.22 μm filter membrane for LC-mass spectrometry determination. The sample extracts were analyzed by UPLC-ESI-MS/MS system. Phytohormone contents were detected by MetWare (http://www.metware.cn) based on the AB Sciex QTRAP 6500 LC-MS/MS platform (Yang et al., 2023; Li et al., 2024).

### 2.4 Widely targeted metabolomics identification and quantitative analysis of non-volatile compounds

Tea samples were placed in a Scientz-100 F freeze-dryer for vacuum freeze-drying, and ground (30 Hz, 1.5 min) to powder using a grinder (MM 400, Retsch). 50 mg of sample powder was weighed, and 1,200 μL of 70% methanol-water internal standard extract pre-cooled at -20°C was added (less than 50 mg was added to 1,200 μL of extractant per 50 mg of sample), vortexed every 30 min for 6 times. After centrifugation (12,000 r·min^-1^,3 min), the supernatant was extracted, filtered with a microporous membrane (0.22 μm pore size), and stored in an injection bottle for UPLC-MS/MS analysis.

The sample extracts were analyzed using an UPLC-ESI-MS/MS system (UPLC, ExionLC™ AD, https://sciex.com.cn/) and Tandem mass spectrometry system (https://sciex.com.cn/) by MetWare (http://www.metware.cn) (Chen et al., 2013). The analytical conditions were as follows, UPLC: column, Agilent SB-C18 (1.8 μm, 2.1 mm×100 mm); The mobile phase consisted of solvent A, pure water with 0.1% formic acid, and solvent B, acetonitrile with 0.1% formic acid. Sample measurements were performed with a gradient program that employed the starting conditions of 95% A, 5% B. Within 9 min, a linear gradient to 5% A, 95% B was programmed, and a composition of 5% A, 95% B was kept for 1 min. Subsequently, a composition of 95% A, 5.0% B was adjusted within 1.1 min and kept for 2.9 min. The flow velocity was set as 0.35 mL per minute; The column oven was set to 40°C; The injection volume was 2 μL. The effluent was alternatively connected to an ESI-triple quadrupole-linear ion trap (QTRAP)-MS.

Mass spectrum scans were acquired using both the Linear Ion Trap (LIT) and Triple Quadrupole (QqQ) modes of a hybrid QqQ LIT Mass Spectrometer (Q TRAP ^®^) (AB6500 Q TRAP^®^ UPLC/MS/MS system) with an ESI Turbo ion spray interface, and both cation and anion modes were controlled by Analyst 1.6.3 software (AB Sciex, MA, USA). The ESI source operation parameters were as follows: source temperature 500°C; ion spray voltage (IS) 5500 V (positive ion mode)/-4500 V (negative ion mode); ion source gas I (GSI), gas II(GSII), curtain gas (CUR) was set at 50, 60, and 25 psi, respectively; the collision-activated dissociation (CAD) was high. QQQ scans were acquired as MRM experiments with collision gas (nitrogen) set to medium. DP (declustering potential) and CE (collision energy) for individual MRM transitions was done with further DP and CE optimization. A specific set of MRM transitions were monitored for each period according to the metabolites eluted within this period.

### 2.5 Transcriptomic analysis

Total RNA of the above test samples was extracted using a Total RNA Extraction Kit (TIANGEN Biotech, Beijing, China). RNA integrity was detected by RNA Nano 6000 Assay Kit of Bioanalyzer 2,100 system (Agilent Technologies, CA, USA). The purity of RNA was detected by NanoPhotometer spectrophotometer (IMPLEN, CA, USA). RNA concentration was measured using the QubitR RNA Assay Kit in the QubitR 2.0 Flurometer (Life Technologies, CA, USA) to ensure the quality of the extracted RNA for subsequent sequencing. The cDNA was synthesized using MMLV reverse transcriptase RNAase (Promega), and the sequencing library was constructed using NEBNextRUltraTMRNA Library Prep Kit for lluminaR (NEB, USA), and then sequenced on the Illumina sequencing platform (Illumina Novaseq 6000) by MetWare (http://www.metware.cn). Clean reads are obtained by removing reads containing adapters and Poly-N sequences and low-quality reads from the raw data using the software fastq v0.23.2 (Chen et al., 2018). It is assembled using Trinity (https://github.com/trinityrnaseq/trinityrnaseq) and clustered to remove redundancy (Grabherr et al., 2011). DIAMOND BLASTX was used to compare the de-redundant transcript sequence with the KEGG, NR, Swiss-Prot, GO, COG/KOG, TrEMBL databases, and the amino acid sequence was compared with the Pfam database to obtain the annotation information of the transcript in the seven databases (Buchfink et al., 2015). FeatureCounts v1.6.2 was used to calculate the number of reads mapped to each gene, and the number of fragments per kilobase per million (FPKM, fragments per kilobase per million) of transcript sequences sequenced per million base pairs is calculated to characterize the abundance of gene transcripts. DESeq2 v1.22.1/edgeR v3.24.3 was used to analyze the differential expression between the two groups (Love et al., 2014). The p-value was corrected using the Benjamini & Hochberg method (Benjamini and Hochberg, 1995). The |log2FoldChange|≥1 and FDR≤ 0.05 were used as criteria to determine the significance of differentially expressed genes. The enrichment analysis was performed using the hypergeometric test. For KEGG, the hypergeometric distribution test was performed using the pathway as the unit of analysis (Zhang et al., 2014).

### 2.6 Data analysis

Data was analyzed using Microsoft 365 Excel software (Microsoft, USA). Duncan’s test was used to determine significant differences between groups. Unsupervised principal component analysis (PCA) was performed using the statistical function prcomp in R v3.5.0 (www.r-project.org). Supervised multiple regression orthogonal partial least squares discriminant analysis (OPLS-DA) was performed using ropls v1.19.8 in R. Transcription factor prediction was performed using the PlantTFDB database (https://planttfdb.gao-lab.org/). Transcription factor binding sites were predicted using TBtools-II v2.096. Promoter cis-acting element analysis was performed using the PlantCare database (https://bioinformatics.psb.ugent.be/). Trend analysis was performed using the OmicShare online tool (https://www.omicshare.com/tools/) for analysis. *P*<0.05 was considered statistically significant. The number of trends is chosen to be 20. KEGG enrichment analysis, K-means clustering analysis and WGCNA were performed using the MetWare Cloud platform (https://cloud.metware.cn/).

## 3 Results

### 3.1 The content of GA in buds and leaves of tea shoots at different developmental stages decreased gradually

**Figure 1:**
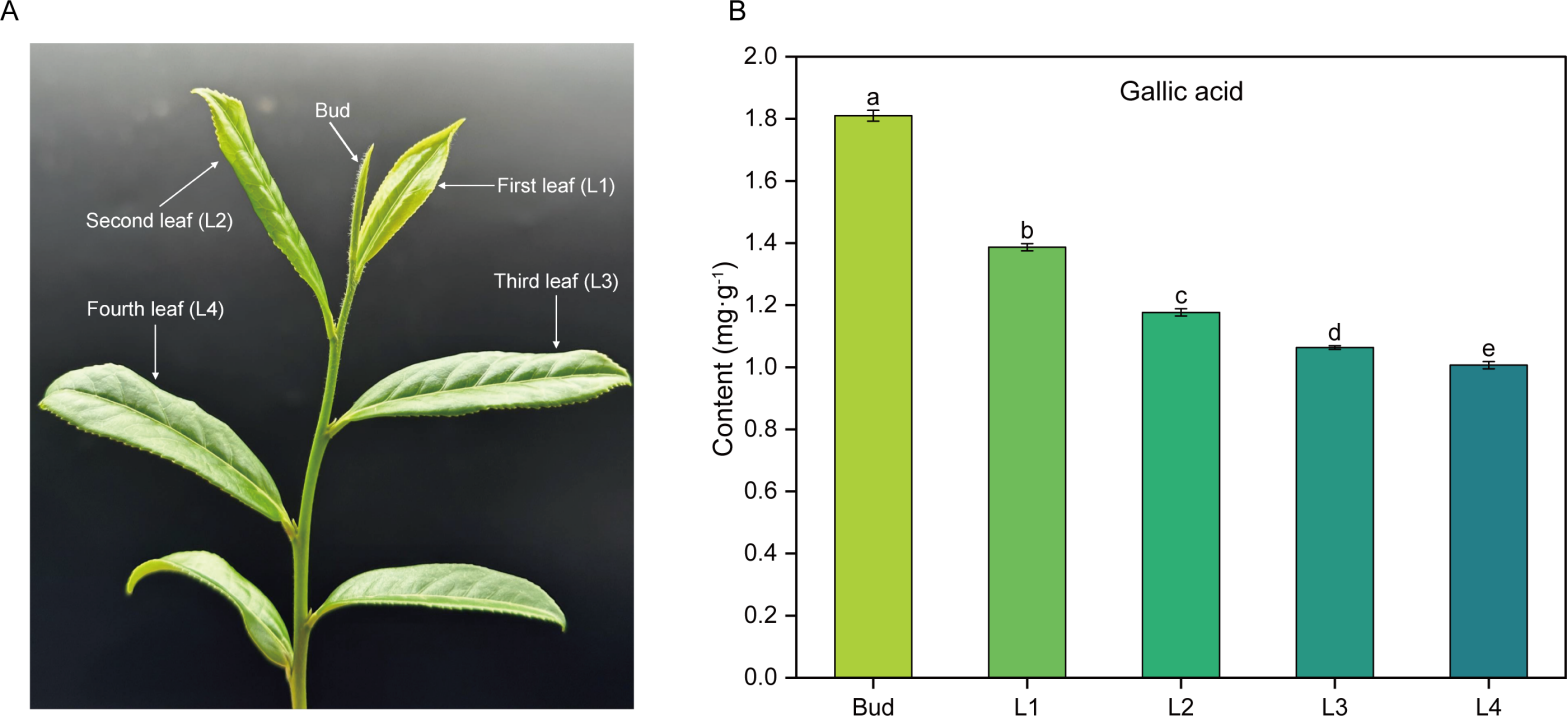
Phenotype of tea shoots and GA content in buds and leaves at different developmental stages. **(A)** Phenotype of tea shoots. **(B)** GA content in buds and leaves of tea shoots at different developmental stages. Bud: the apical bud; L1: the first leaf; L2: the second leaf; L3: the third leaf; L4: the fourth leaf.

High performance liquid chromatography (HPLC) was used to determine the content of GA in the buds and leaves of five different development stages of tea shoots (**Figure 1**). The results showed that GA content gradually decreased from bud to the fourth leaf, and there was a significant difference (*P*<0.05) in GA content among the five samples. Compared with Bud, the content of GA in L1, L2, L3 and L4 decreased by 23.45%, 34.88%, 41.28% and 44.52%, respectively.

### 3.2 Accumulation of GA in buds and leaves of tea shoots at different developmental stages was related to the changes of endogenous hormone components

**Figure 2:**
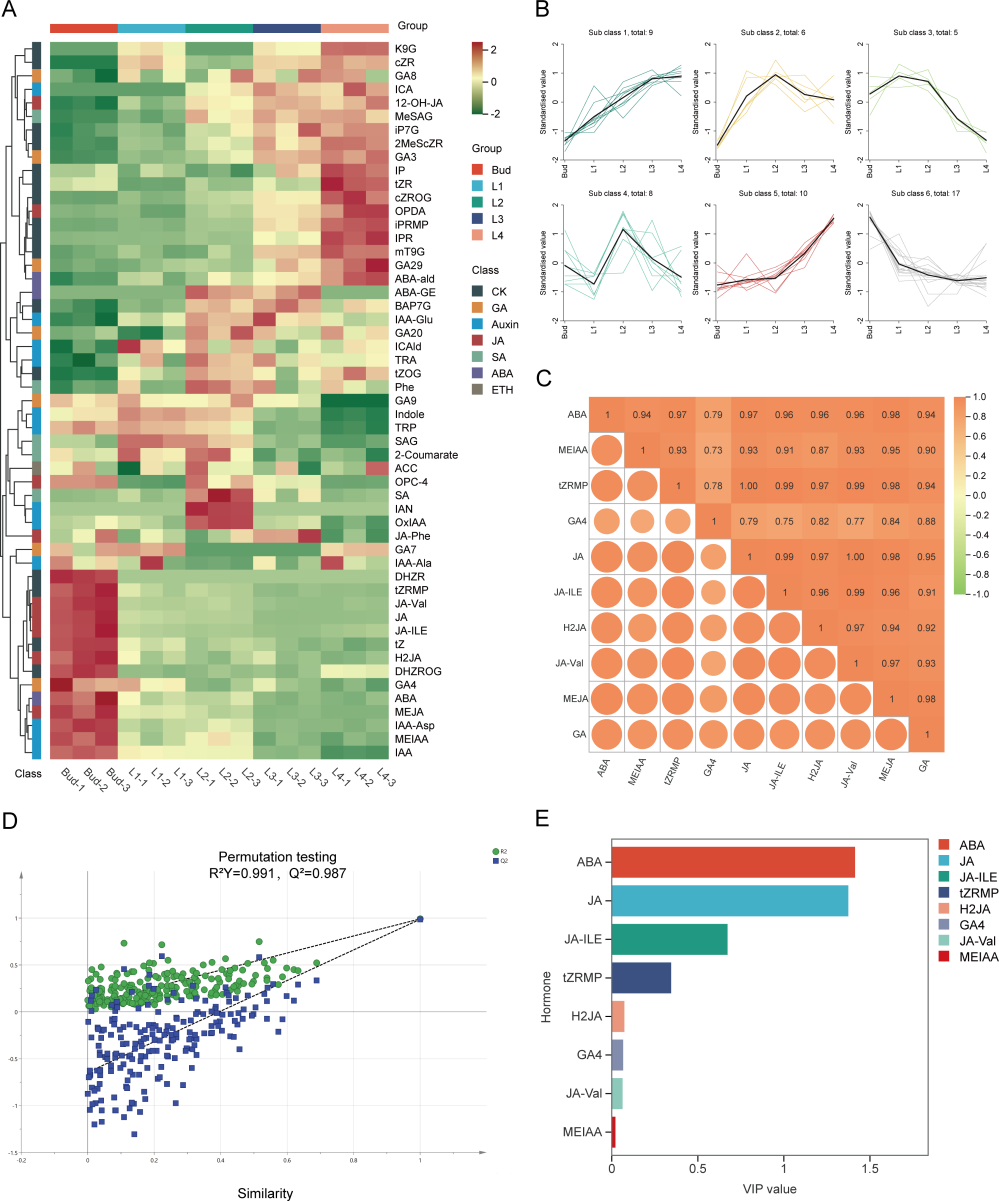
Relationship between endogenous hormone and GA in buds and leaves of tea shoots at different developmental stages. **(A)** Cluster heatmap of endogenous hormone content in buds and leaves of tea shoots at different developmental stages.**(B)** K-means clustering diagram of 53 endogenous hormone content in buds and leaves of tea shoots at different developmental stages. **(C)** Correlation between 9 endogenous hormone components and GA content. **(D)** The cross-validation diagram of the OPLS-DA model of endogenous hormone content and GA content. **(E)** VIP values of 9 endogenous hormone components.

In order to investigate the changes of endogenous phytohormones in the buds and leaves of tea shoots at different developmental stages, the contents of cytokinin, auxin, jasmonic acid, gibberellin, salicylic acid, abscisic acid and ethylene in the samples were determined by HPLC-MS/MS. A total of 53 endogenous hormones were detected in the samples, and most of the plant hormone components showed different expression patterns (**Figure 2A**). K-means clustering analysis of 53 endogenous hormones and GA contents (**Figure 2B**) showed that the variation patterns of 16 endogenous hormone components such as ABA, MEIAA, IAA, IAA-Ala, IAA-Asp, DHZR, tZRMP, DHZROG, tZ, GA4, GA7, JA, JA-ILE, H2JA, JA-Val and MEJA in Sub Class 6 were consistent with the variation of GA content. It indicated that the differential accumulation of GA in buds and leaves of tea shoots was closely related to the changes of endogenous hormones such as jasmonic acid, abscisic acid, auxin, cytokinin, and gibberellin. Pearson correlation analysis showed that the contents of JA, JA-ILE, H2JA, JA-Val, MEJA, ABA, MEIAA, tZRMP, and GA4 were significantly positively correlated with GA content (r>0.85, *P*<0.05) (**Figure 2C**). Therefore, it is speculated that these nine endogenous hormone components are involved in the development of tea shoots on the one hand, and on the other hand, they can directly or indirectly regulate the biosynthesis of GA during the development of buds and leaves. The OPLS-DA model (R^2^X=0.942, R^2^Y=0.991) was further established by combining 53 endogenous hormone components and GA content. The model had a 99.10% interpretation rate for the change of GA content with endogenous hormone content, and Q^2^ = 0.987(>0.5), indicating that the prediction ability of the model was better (**Figure 2D**). The VIP values of ABA and JA were both greater than 1 in the nine endogenous hormone components (**Figure 2E**). Based on the above analysis, it was speculated that the changes of JA and ABA contents had a more important effect on the differential accumulation of GA content during the development of tea shoots.

### 3.3 Metabolic mechanism of differential accumulation of GA content in buds and leaves of tea shoots at different developmental stages

**Figure 3:**
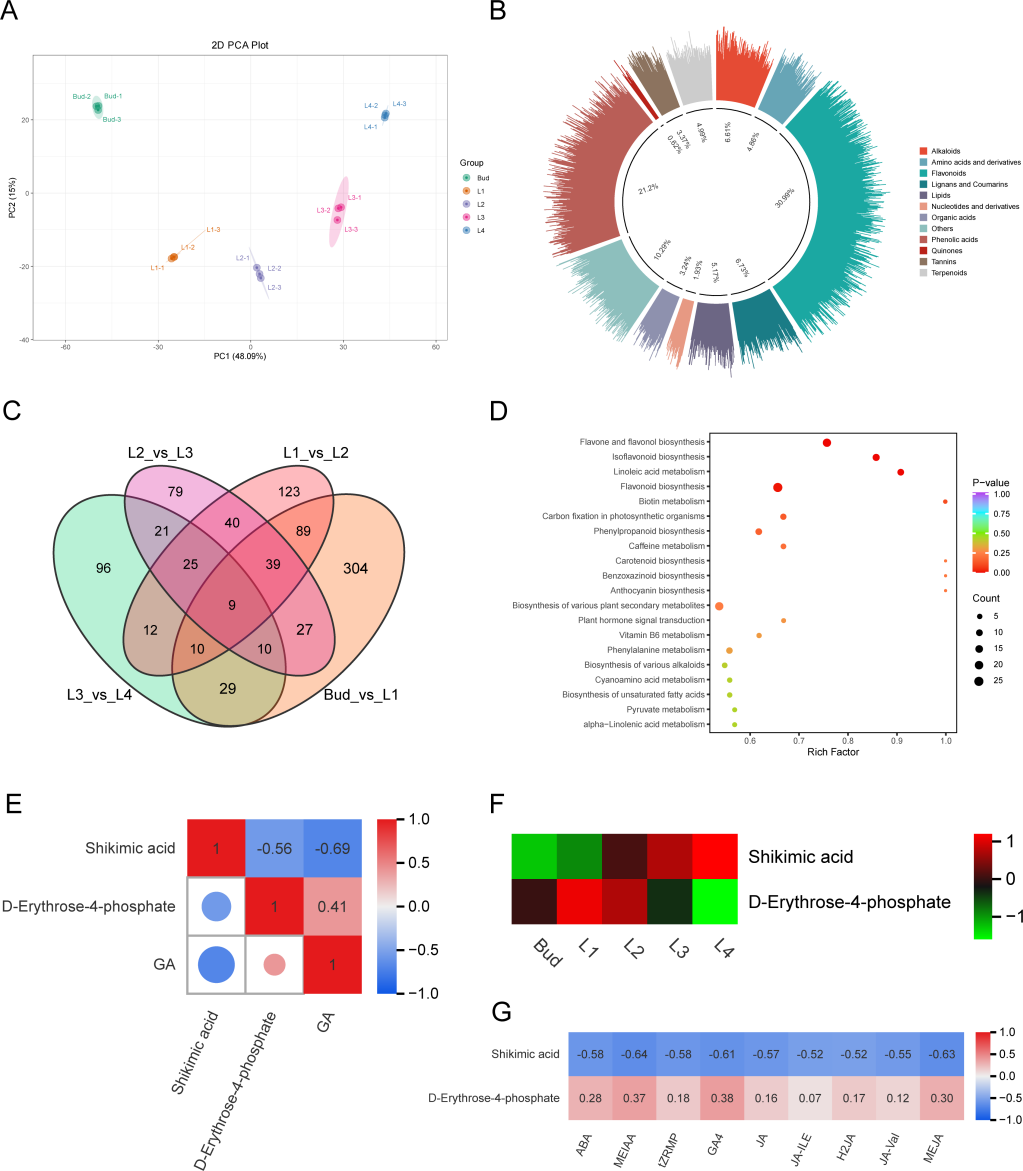
Analysis of metabolites in buds and leaves of tea shoots at different developmental stages. **(A)** PCA diagram of all metabolites. **(B)** Classification of differential accumulated metabolites. **(C)** Veen plot of differential accumulated metabolites. **(D)** KEGG enrichment bubble diagram of differential accumulated metabolites. **(E)** Correlation between differential accumulated metabolites related to GA biosynthesis and GA content. **(F)** Expression heatmap of differential accumulated metabolites related to GA biosynthesis. **(G)** Correlation between the content of differential accumulated metabolites related to GA biosynthesis and the content of 9 endogenous hormones.

In order to explore the metabolic mechanism underlying the differential accumulation of GA content in buds and leaves of tea shoots at different developmental stages, the relative content of non-volatile metabolites in the samples was determined by LC-ESI-MS/MS, and a total of 2,590 metabolites were identified. PCA analysis showed that there was a significant separation between the five groups (**Figure 3A**). The first principal component accounted for 48.09% of the metabolic differences between the samples, separating the five different developmental stage samples significantly, and the second principal component accounted for 15.00% of the metabolic differences between the samples, separating Bud and L4 from the other samples significantly. According to the variable importance in project (VIP) score obtained by the OPLS-DA model, the differentially accumulated metabolites (DAMs) in the five groups of samples were identified with VIP>1 and fold chang≥2 or fold change ≤ 0.5 as the screening conditions. A total of 1,604 DAMs were identified, including 497 flavonoids (30.99%), 340 phenolic acids (21.20%), 78 amino acids and their derivatives (4.86%), 31 nucleotides and their derivatives (1.93%), 10 quinones (0.62%), 108 lignans and coumarins (6.73%), 54 tannins (3.37%), 106 alkaloids (6.61%), 80 terpenoids (4.99%), 52 organic acids (3.24%), 83 lipids (5.17%), and 165 other compounds (10.29%) (**Figure 3B**). It can be seen that phenolic acids are the main DAMs in buds and leaves of tea shoots at different developmental stages. The Venn diagram (**Figure 3C**) showed that there were 9 common DAMs in the four comparison groups of Bud vs L1, L1 vs L2, L2 vs L3, and L3 vs L4, and there were 304, 123, 79 and 96 unique DAMs, respectively. It shows that the greater the difference in leaf development stage, the more the number of DAMs; the difference of metabolic level between Bud and L1 was the largest. KEGG enrichment analysis showed that the DAMs were significantly enriched in the pathways of biosynthesis of various plant secondary metabolites (ko00999) and plant hormone signal transduction (ko04075), indicating that the metabolites of phenolic acids and hormone-related metabolites varied greatly among different samples (**Figure 3D**). We identified two GA biosynthesis-related metabolites D-erythrose-4-phosphate and shikimic acid in shikimic acid pathway from the DAMs. D-erythritol-4-phosphate, a substrate upstream of the shikimic acid pathway, its content was positively correlated with GA content (r=0.41), and gradually decreased From L1 to L4; shikimic acid, a competitive metabolite of GA, gradually increased with the development of buds and leaves, and its content was significantly negatively correlated with GA content (r=-0.69, *P*<0.05) (**Figures 3E, F**). Correlation analysis showed that the content of D-erythritol-4-phosphate was positively correlated with the contents of ABA, MEIAA, tZRMP, GA4, JA, JA-ILE, H2JA, JA-Val and MEJA (r=0.07~0.38), while the content of shikimic acid was significantly negatively correlated with the contents of ABA, MEIAA, tZRMP, GA4, JA, JA-ILE, H2JA, JA-Val and MEJA (r<-0.5, *P*<0.05) (**Figure 3G**). These results suggested that a decrease in the content of D-erythrose-4-phosphate and an increase in the content of shikimic acid shadowed the accumulation of GA during the development of buds and leaves of tea shoots.

### 3.4 Transcriptome analysis of buds and leaves at different developmental stages of tea shoots

**Figure 4:**
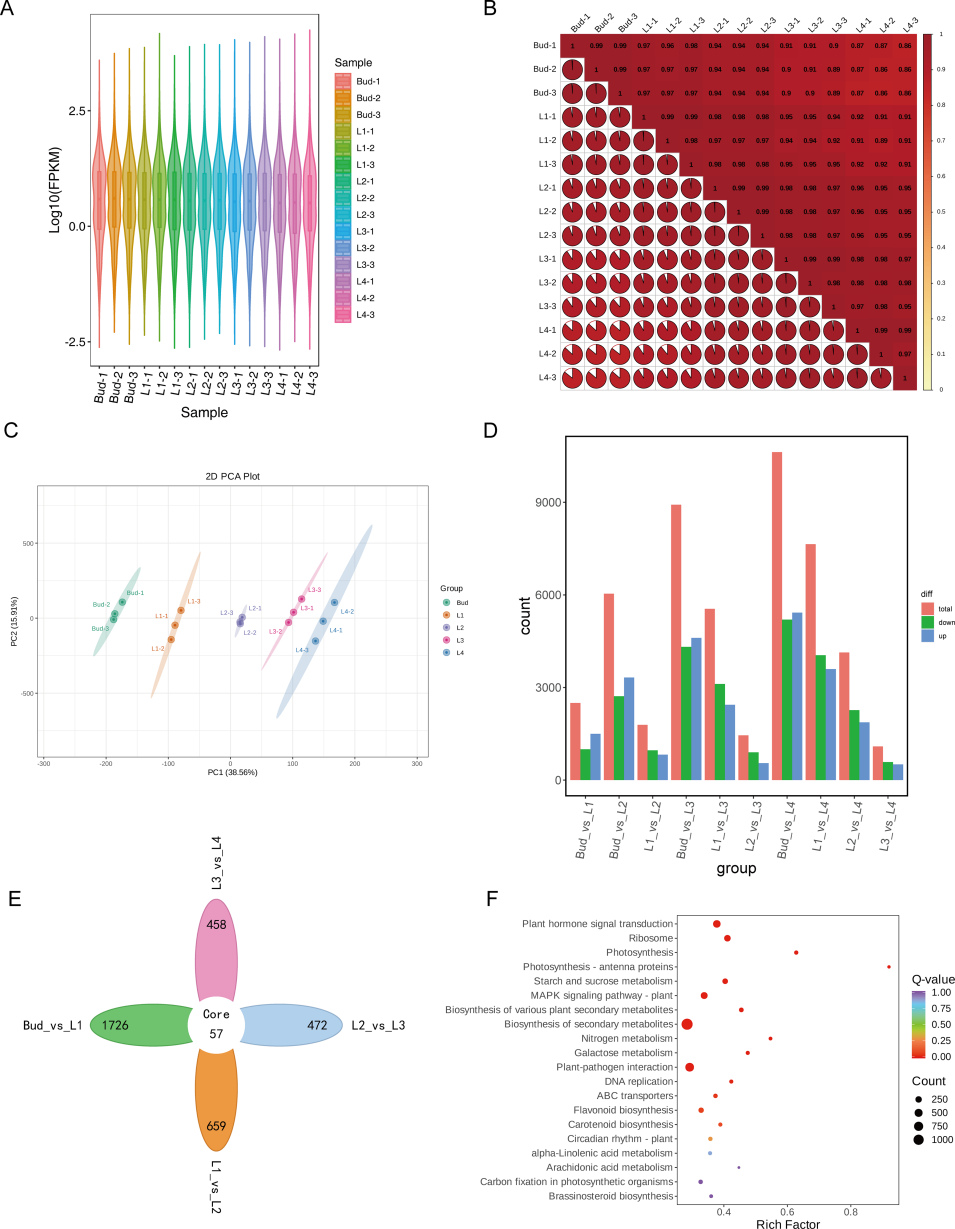
Transcriptome analysis of buds and leaves at different developmental stages of tea shoots. **(A)** Violin plot of gene expression in samples. **(B)** Correlation between samples. **(C)** PCA analysis of all samples. **(D)** Number of differentially expressed genes in 10 comparison groups. **(E)** Veen diagram of differentially expressed genes in comparison groups of Bud vs L1, L1 vs L2, L2 vs L3 and L3 vs L4. **(F)** KEGG enrichment bubble diagram of differentially expressed genes.

To further investigate the molecular mechanism of differential accumulation of GA in the buds and leaves of tea shoots at different developmental stages, transcriptome sequencing was performed on 15 samples from five groups using the Illumina sequencing platform. A total of 114.9 Gb of Clean Data was obtained by filtering to remove low-quality fragments. The Clean Data of each sample was above 6 Gb, and a total of 766,009,080 Clean reads were obtained. The number of clean reads in each sample was between 44,844,750 and 580,129,456, Q20 was greater than 96%, Q30 was greater than 91%, and the overall sequencing error rate of the sample was less than 0.03%, indicating that the overall sequencing accuracy was high. The GC content was about 45%, indicating that the sequencing process instrument was stable. The filtered data were aligned to the reference genome of tea plant ‘Huangdan’ using HISAT2, and the alignment rate was above 92.04% (**Supplementary Table 1**). A total of 41,702 expressed genes were detected, including 31,743 known genes and 9,959 new genes. The violin plot intuitively shows the probability density and distribution of each set of data (**Figure 4A**). The results of correlation analysis between samples showed that there was a high correlation between three repeated samples in each group (**Figure 4B**). PCA analysis showed that the five groups of samples were significantly separated, and principal component 1 and principal component 2 explained 38.56% and 15.91% of the total variance, respectively, indicating that the gene expression of the five groups of samples at different developmental stages was significantly different (**Figure 4C**). A total of 12,850 differentially expressed genes (DEGs) were identified from 10 comparison groups of Bud vs L1, Bud vs L2, L1 vs L2, Bud vs L3, L1 vs L3, L2 vs L3, Bud vs L4, L1 vs L4, L2 vs L4, L3 vs L4, with |log_2_Fold Change|≥1 and FDR<0.05 as the condition. Among them, 2,498 DEGs were detected in the Bud vs L1 comparison group (1,499 up-regulated and 999 down-regulated); in the L1 vs L2 comparison group, 1,787 DEGs were detected (822 up-regulated and 965 down-regulated); in the L2 vs L3 comparison group, 1,448 DEGs were detected, including 549 up-regulated genes and 899 down-regulated genes; in the L3 vs L4 comparison group, 1,091 DEGs were detected, including 508 up-regulated genes and 583 down-regulated genes (**Figure 4D**). The Venn diagram showed that there were 57 common DEGs in the four comparison groups of Bud vs L1, L1 vs L2, L2 vs L3 and L3 vs L4, and the number of unique DEGs in each group were 1726, 659, 472 and 458, respectively (**Figure 4E**). It can be seen that the number of DEGs gradually decreased during the development of buds and leaves of tea shoots. Among the five groups of samples from different leaf positions, the largest differences in gene expression levels were found in Bud and L1, which was consistent with the metabolite assay. KEGG enrichment analysis showed that the DEGs were significantly enriched in biosynthesis of various plant secondary metabolites (ko00999), plant hormone signal transduction (ko04075), biosynthesis of secondary metabolite (ko01100), and flavonoid biosynthesis (ko00941) pathways (**Figure 4F**), it shows that during the development of tea shoots, the biosynthesis of secondary metabolites such as phenolic acids and flavonoids and the expression of genes related to plant hormone signaling pathways in tea shoots were significantly different.

### 3.5 Weighted gene co-expression network analysis

**Figure 5:**
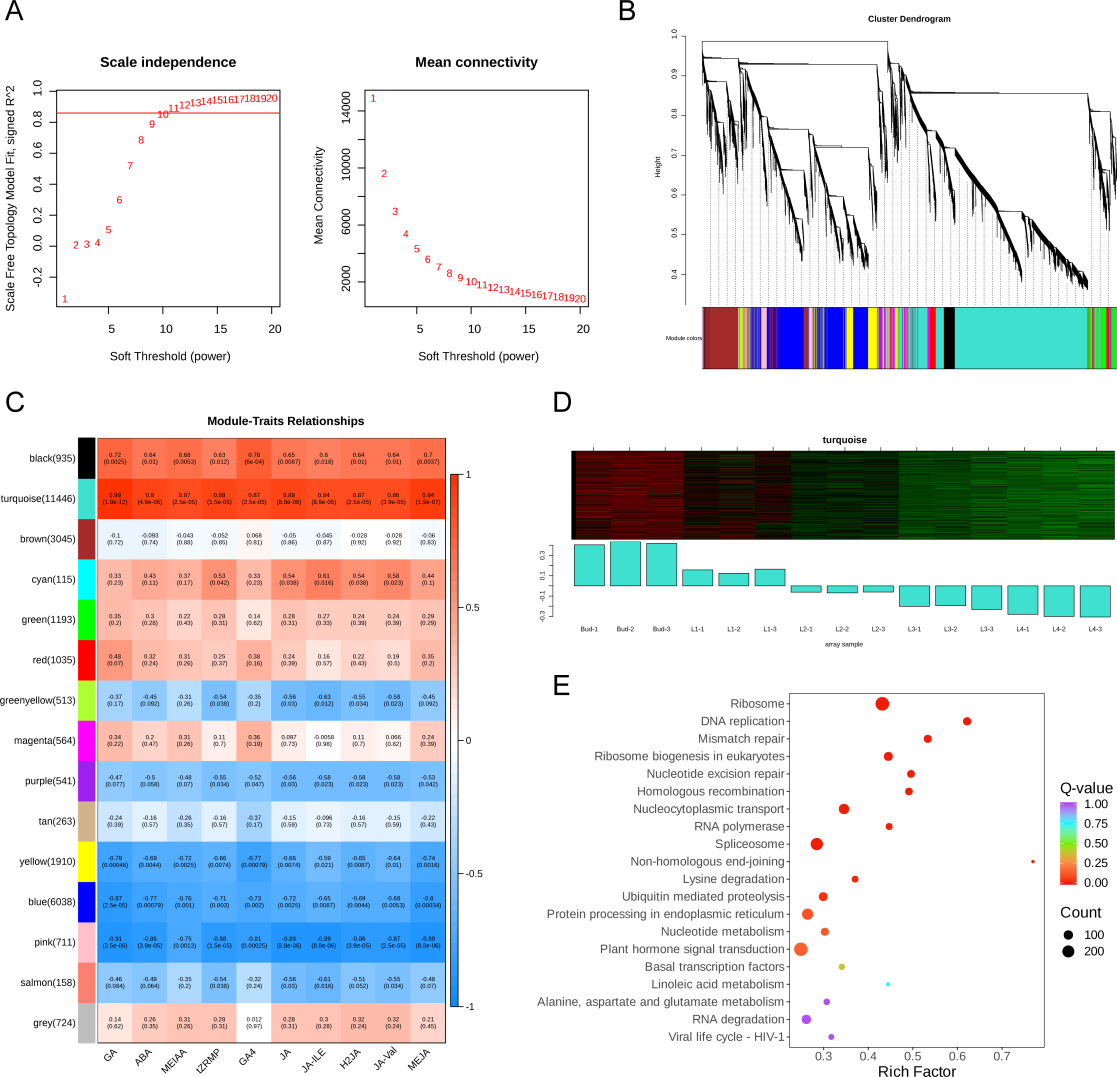
The results of WGCNA. **(A)** Analysis of network topology for various soft-thresholding powers. The left panel shows the scale-free fit index (y-axis) as a function of the soft-thresholding power (x-axis). Power 10 was chosen because the fit index curve flattened out upon reaching a high value (> 0.85). The right panel displays the mean connectivity (degree, y-axis) as a function of the soft-thresholding power (x-axis). **(B)** The cluster dendrogram and color display of co-expression network modules for all genes. The short vertical line corresponded to a gene and the branches corresponded to the co-expressed genes. **(C)** Correlation matrix of module eigengene values obtained from WGCNA. 15 modules were identified, and each module eigengene was tested for correlation with trait. Within each cell, upper values are correlation coefficients between module eigengene and the traits; lower values are the corresponding *p*-value. Color-coded by relevance based on a color legend. Blue rectangles represent negative correlations between modules and traits, and red rectangles represent positive correlations between modules and traits. **(D)** Gene expression heatmap in turquoise module. **(E)** KEGG enrichment bubble diagram of genes in turquoise module.

In order to gain insight into the mechanism of endogenous hormones regulating GA biosynthesis, 29,191 genes were selected for WGCNA analysis after filtering the genes with low variation in the RNA-Seq expression matrix of five groups of samples. The β value (soft power=10) was selected to construct a weighted gene network (**Figure 5A**) and the 29,191 genes were divided into 15 different colored co-expression network modules according to the dynamic shear method (**Figure 5B**). The correlation analysis between the content of GA and 9 endogenous hormone components and 15 gene modules showed that the turquoise module was significantly positively correlated with the content of GA and 9 endogenous hormone components (**Figure 5C**). With the development of the bud and leaves, the genes in the turquoise module were significantly down-regulated (**Figure 5D**) and the results of KEGG analysis showed that the genes within the turquoise module were significantly enriched in the pathways of phytohormone signal transduction (ko04075), ribosome (ko03010), and DNA replication (ko03030) (**Figure 5E**).

#### 3.5.1 Analysis of co-expressed structural genes regulating GA biosynthesis in buds and leaves of tea shoots at different developmental stages

**Figure 6:**
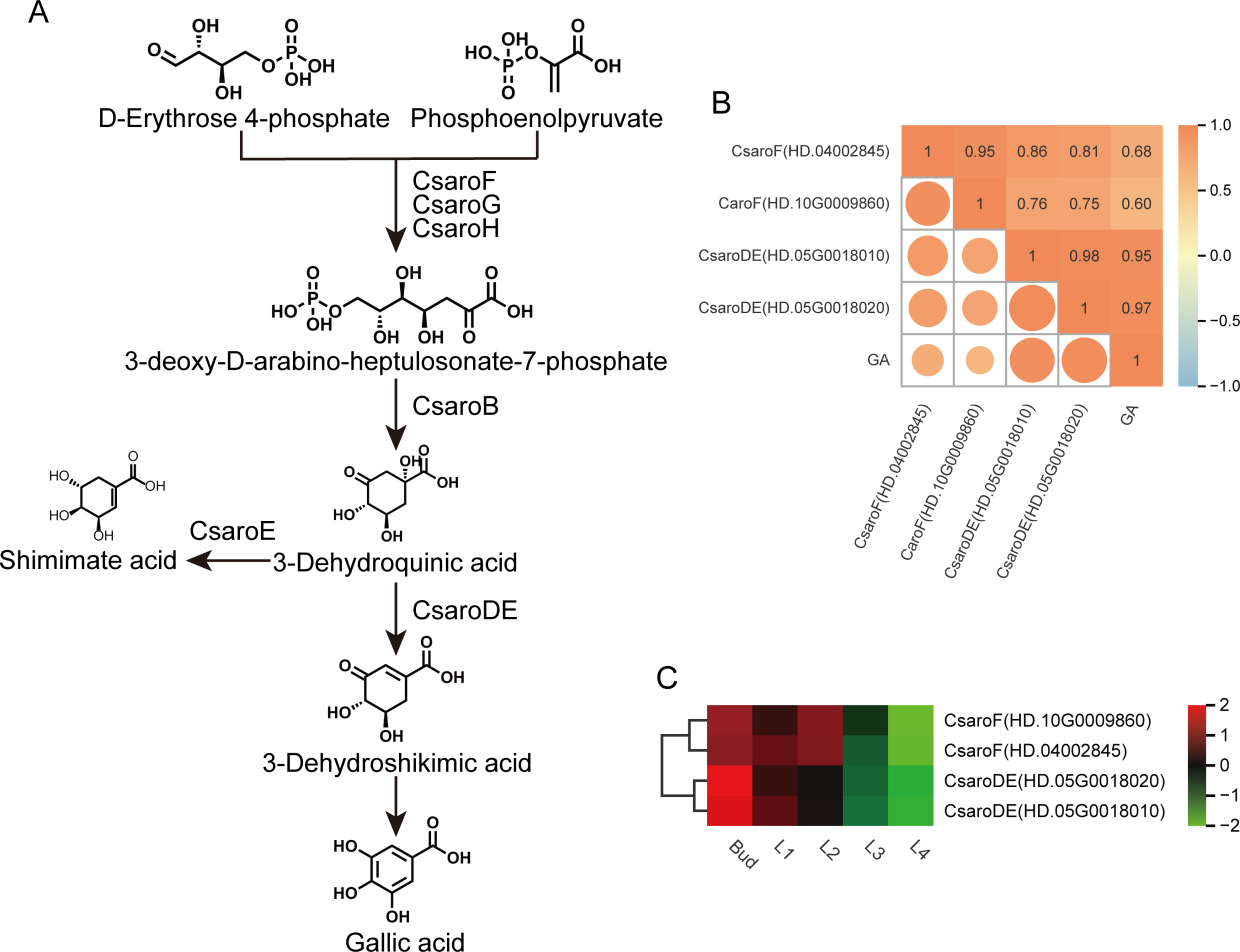
GA biosynthesis pathway and the expression of related structural genes. **(A)** GA biosynthesis pathway. **(B)** Correlation between the expression of structural genes related to GA biosynthesis and GA content. **(C)** Heatmap of the expression of GA biosynthesis-related structural genes.

Based on the results of KEGG enrichment analysis and gene function annotation, four genes related to GA biosynthesis in shikimic acid pathway were identified from the turquoise module (**Figure 6A**), namely *CsaroF*(HD.04002845), *CsaroF*(HD.10G0009860), *CsaroDE*(HD.05G0018010) and *CsaroDE*(HD.05G0018020). The expression levels of these four genes were significantly positively correlated with GA content (r≥0.6, *P*<0.05) (**Figure 6B**). The correlation coefficients of *CsaroDE*(HD.05G0018010) and *CsaroDE*(HD.05G0018020) with GA content were 0.95 and 0.97 respectively, and the *P* value was the smallest (*P*=4.44×10^-8^, *P*=4.97×10^-9^). Therefore, it can be explained that the down-regulated expression of *CsaroF*(HD.04002845), *CsaroF*(HD.10G0009860), *CsaroDE*(HD.05G0018010) and *CsaroDE*(HD.05G0018020) during the development of tea shoots (**Figure 6C**) inhibited the biosynthesis of GA, and *CsaroDE*(HD.05G0018010) and *CsaroDE*(HD.05G0018020) were the key genes regulating GA biosynthesis.

#### 3.5.2 Analysis of co-expressed transcription factors regulating GA biosynthesis in buds and leaves of tea shoots at different developmental stages

**Figure 7:**
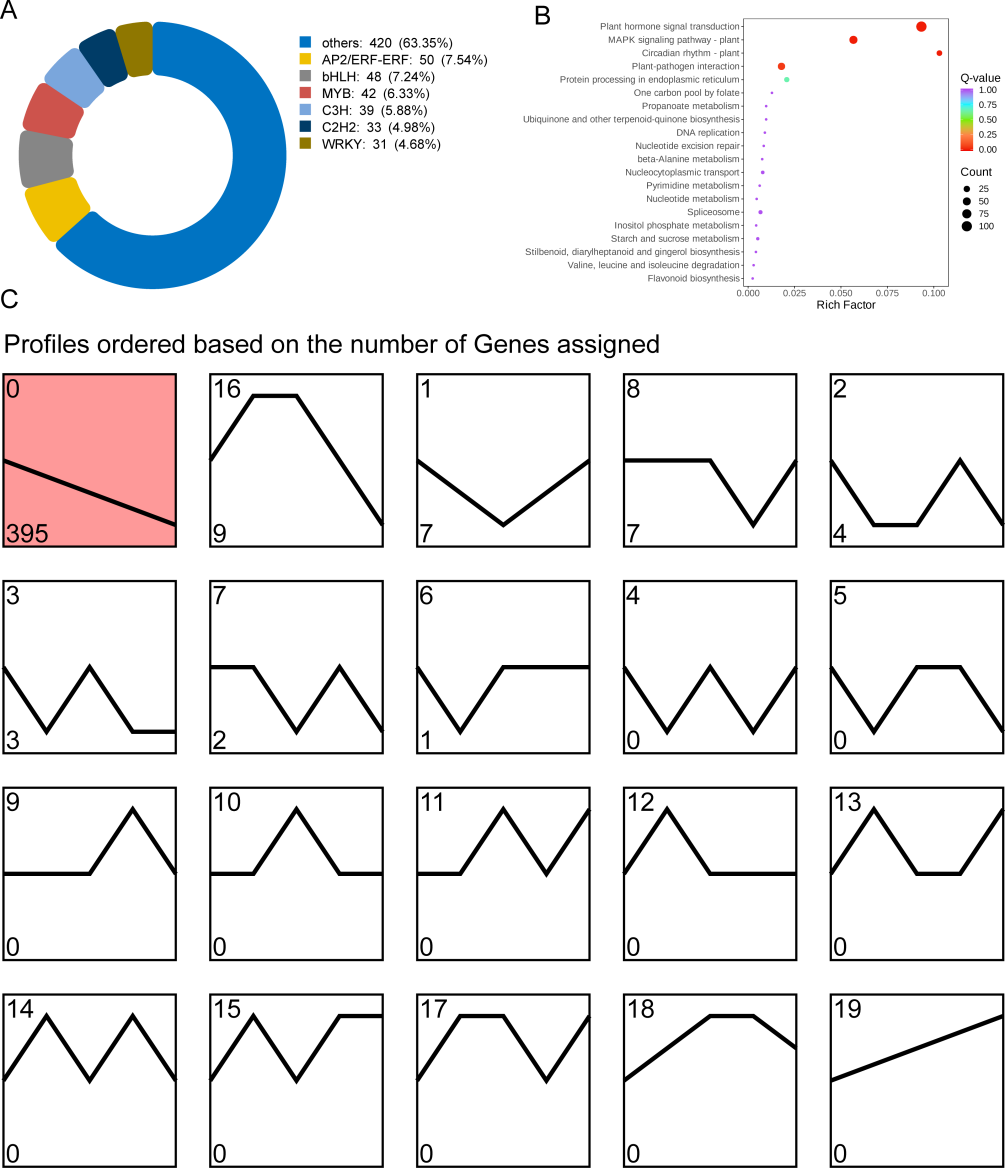
Analysis of co-expressed transcription factors regulating GA biosynthesis. **(A)** Classification of 663 transcription factors co-expressed with GA. **(B)** KEGG enrichment bubble diagram of transcription factors. **(C)** Trend analysis of transcription factors in turquoise module.

In order to investigate the transcription factors regulating GA biosynthesis during the development of tea shoots, a total of 663 transcription factors were identified from the turquoise module, belonging to 55 transcription factor families, of which the families of AP2/ERF-ERF(50), bHLH(48), MYB(42), C3H(39), C2H2(33) and WRKY(31) with high numbers of transcription factors (**Figure 7A**); KEGG enrichment analysis showed that transcription factors were significantly enriched in pathways such as plant hormone signal transduction (ko04075), MAPK signaling pathway-plant (ko04016), circadian rhythm-plant (ko04712), protein processing in endoplasmic reticulum (ko04141) and plant-pathogen interaction (ko04626) (**Figure 7B**). Based on the expression trend analysis, 663 transcription factors were divided into 20 modules. Among them, the expression trend of 395 transcription factors in Profile 0 was consistent with the change pattern of GA content (**Figure 7C**, **Supplementary Table 2**), suggesting that these transcription factors were involved in the regulation of GA biosynthesis.

#### 3.5.3 Analysis of co-expressed plant hormone signal transduction factors regulating GA biosynthesis in buds and leaves of tea shoots at different developmental stages

**Figure 8:**
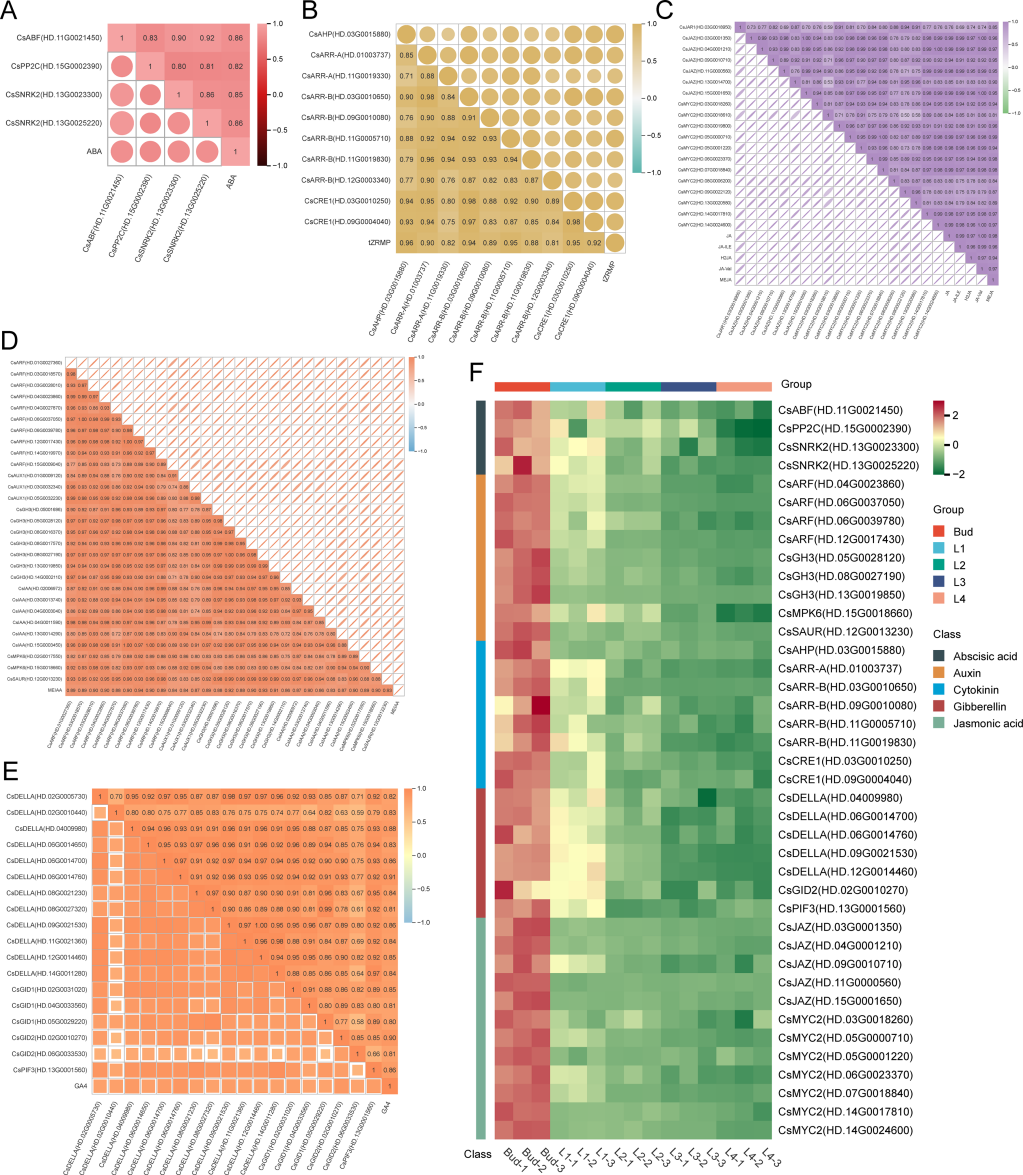
Analysis of plant hormone signal transduction factors in turquoise module. **(A)** Correlation between the expression of ABA signal transduction factors and ABA content. **(B)** Correlation between the expression of Cytokinin signal transduction factors and tZRMP content. **(C)** Correlation between the expression of Jasmonic acid signal transduction factors and JA, JA-ILE, H2JA, JA-Val and MEJA content. **(D)** Correlation between the expression of Auxin signal transduction factors and MEIAA content. **(E)** Correlation between the expression of Gibberellin signal transduction factors and GA4 content. **(F)** Heatmap of the expression of 40 hormone signal transduction factors.

A total of 13 abscisic acid signaling pathway genes were identified from the turquoise module, of which 3 genes were significantly correlated with ABA content (r>0.8, *P*<0.05), including 1 *CsABF*, 2 *CsSNRK2*, and 1 *CsPP2C* (r>0.8, *P*<0.05) (**Figure 8A**); 27 cytokinin signaling pathway genes were identified, of which 8 genes were significantly correlated with tZRMP content (r>0.9, *P*<0.05), including 1 *CsAHP*, 1 *CsARR-A*, 4 *CsARR-B*, and 2 *CsCRE1* (**Figure 8B**); 25 jasmonic acid signaling pathway genes, of which 12 genes were all significantly correlated with the content of 5 jasmonic acid components (r>0.9, *P*<0.05), including 1 *CsJAR1*, 6 *CsJAZ*, and 12 *CsMYC2* (**Figure 8C**); 53 genes of the auxin signaling pathway, of which 9 genes were significantly correlated with the MEIAA content (r>0.9, *P*<0.05), including 4 *CsARF*, 3 *CsGH3*, 1 *CsMPK6*, and 1 *CsSAUR* (**Figure 8D**); 51 gibberellin signaling pathway genes, of which 7 genes were significantly correlated with GA4 content (r>0.85, *P*<0.05), including 5 *CsDELLA*, 1 *CsGID2*, and 1 *CsPIF3* (**Figure 8E**). The heatmap showed that the expression of 40 plant hormone signal transduction factors in Bud was significantly higher than that in the leaf samples, and gradually decreased from Bud to L4 (**Figure 8F**). It is speculated that these plant hormone signal transduction factors can respond to changes in endogenous hormone content during the development of tea shoot and participate in intracellular signal transduction by binding to specific receptors, thereby regulating the biosynthesis of secondary metabolites in buds and leaves.

#### 3.5.4 The internal relations between endogenous hormone signal transduction factors, transcription factors and GA biosynthesis structural genes

**Figure 9:**
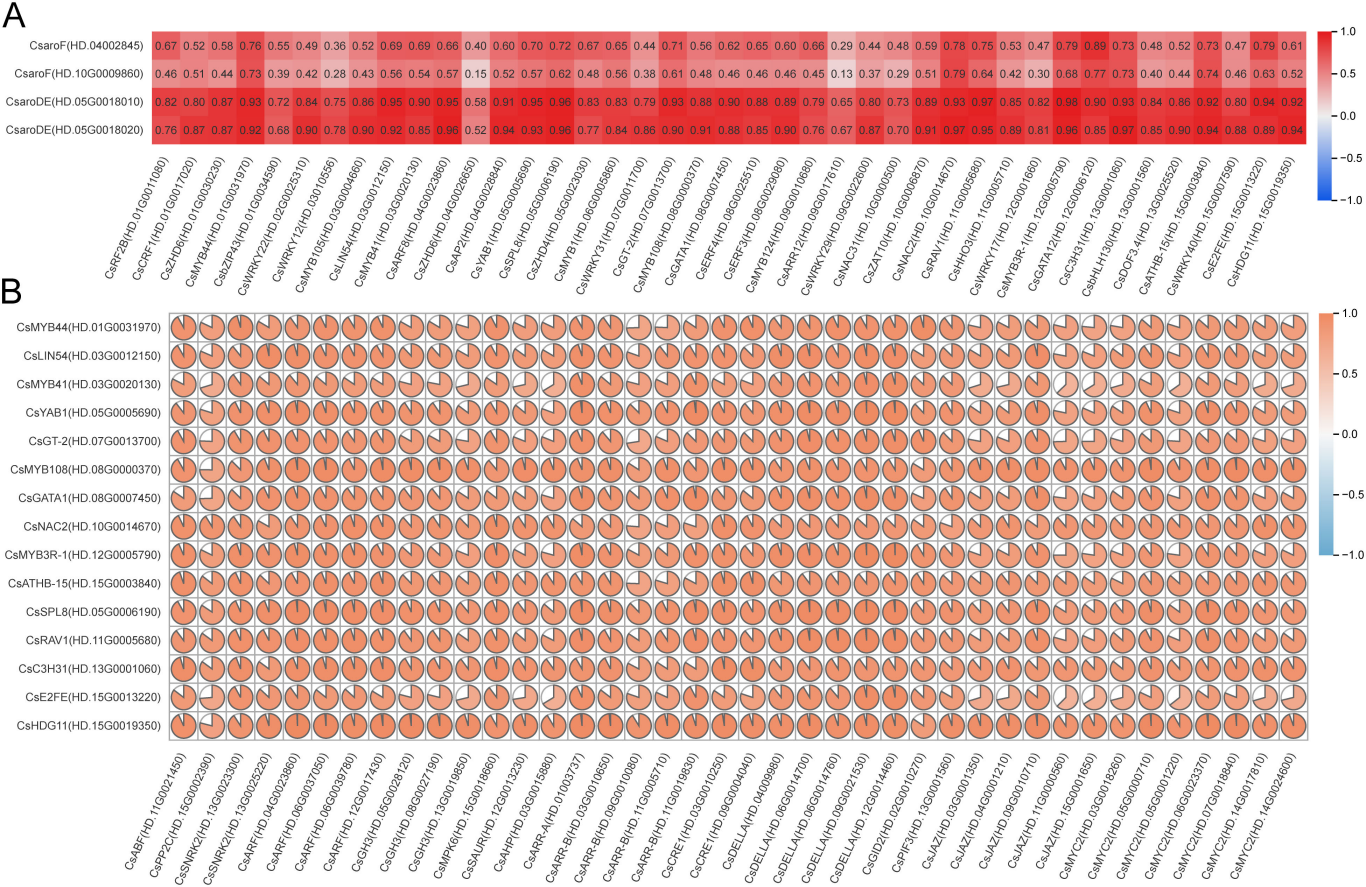
The correlation of GA biosynthesis related transcription factors with GA biosynthesis structural genes and endogenous hormone signal transduction factors. **(A)** Correlation between 41 transcription factors and 4 GA biosynthetic structural genes. **(B)** Correlation between 40 endogenous hormone signal transduction factors and 15 transcription factors.

The transcription factor binding sites of the 4 GA biosynthesis structural genes *CsaroF*(HD.04002845), *CsaroF*(HD.10G0009860), *CsaroDE*(HD.05G0018010) and *CsaroDE*(HD.05G0018020) were predicted using the FIMO motif search tool of the MEME Suite. The results showed that the four structural genes had binding sites with 41 transcription factors in 395 transcription factors (**Supplementary Table 3**). Correlation analysis between 41 transcription factors and 4 structural genes showed that 17 transcription factors such as *CsMYB44* (HD.01G0031970), *CsLIN54* (HD.03G0012150) and *CsMYB41* (HD.03G0020130) were significantly correlated with *CsaroDE*(HD.05G0018010), while *CsMYB44*(HD.01G0031970), *CsLIN54*(HD.03G0012150), *CsARF8*(HD.04G0023860) and other 13 transcription factors were significantly correlated with *CsaroDE*(HD.05G0018020) (r>0.9, *P*<0.05) (**Figure 9A**). Based on the results of correlation analysis and transcription factor binding site prediction, the promoter region of *CsaroDE*(HD.05G0018010) contained the binding sites of 10 transcription factors such as *CcMYB41*(HD.03G0020130) and the expression level was highly correlated with these 10 transcription factors. *CsaroDE*(HD.05G0018020) is highly correlated with 10 transcription factors such as *CsMYB44*(HD.01G0031970) and the promoter region contains the binding sites of these 10 transcription factors. These results showed that 16 transcription factors such as *CsMYB44* (HD.01G0031970), *CsLIN54* (HD.03G0012150), *CsMYB41* (HD.03G0020130) could not only bind to the key structural genes of GA biosynthesis, but also highly correlated with the structural genes of GA biosynthesis. It is speculated that these 16 transcription factors are involved in the regulation of GA biosynthesis during the development of tea shoots.

Cis-acting elements in the promoter region of 16 transcription factors were identified using PlantCare database. The results demonstrated that the promoter regions of 10 transcription factors contained auxin response elements AuxRR-core, TGA-element and TGA-box; the promoter regions of 10 transcription factors contained the abscisic acid response element ABRE; the promoter regions of 10 transcription factors contained gibberellin response elements P-box, TATC-box and GARE-motif, the promoter regions of 4 transcription factors contain cytokinin response element O2-site, and the promoter regions of 13 transcription factors contained MEJA response elements CGTCA-motif and TGACG-motif (**Supplementary Table 4**). It is concluded that these transcription factors can respond to one or several endogenous hormone signals, regulate the expression of structural genes involved in GA biosynthesis, thus affecting the accumulation of GA.

The correlation analysis between these hormone signal transduction factors and transcription factors containing hormone response elements in the promoter region showed that 10 transcription factors were significantly positively correlated with *CsABF*(HD.11G0021450), *CsSNRK2*(HD.13G0023300) and *CsSNRK2*(HD.13G0025220); 10 transcription factors were positively correlated with *CsARF*(HD.04G0023860), *CsARF*(HD.06G0037050), *CsARF*(HD.06G0039780), *CsARF*(HD.12G0017430) and *CsMPK6*(HD.15G001866); *CsMYB44*(HD.01G0031970), *CsMYB41*(HD.03G0020130), *CsGATA1*(HD.08G0007450), *CsC3H31*(HD.13G0001060) were positively correlated with *CsARR-A*(HD.01003737), *CsARR-B*(HD.03G0010650), *CsARR-B*(HD.11G0019830), *CsCRE1*(HD.03G0010250) and *CsCRE1*(HD.09G0004040); 10 transcription factors were significantly positively correlated with 6 gibberellin signal transduction factors, such as *CsDELLA*(HD.04009980), *CsDELLA*(HD.06G0014700), *CsDELLA*(HD.06G0014760), *CsDELLA*(HD.09G0021530), *CsDELLA*(HD.12G0014460) and *CsPIF3*(HD.13G0001560); 13 transcription factors were significantly positively correlated with *CsJAZ*(HD.09G0010710), *CsMYC2* (HD.05G0000710), *CsMYC2*(HD.06G0023370) and *CsMYC2*(HD.07G0018840) (r>0.8, *P*<0.05) (**Figure 9B**). The results indicate that these plant hormone signal transduction factors can respond to changes in endogenous hormone content, activate endogenous hormone signal transduction pathways, regulate the expression of transcription factors, and thus affect the biosynthesis of GA.

### 3.6 The molecular mechanism of endogenous hormones regulating GA biosynthesis during the development of buds and leaves of tea shoots

#### 3.6.1 Endogenous hormone signal transduction factors mediate transcription factors to regulate the expression of GA biosynthetic structural genes

**Figure 10:**
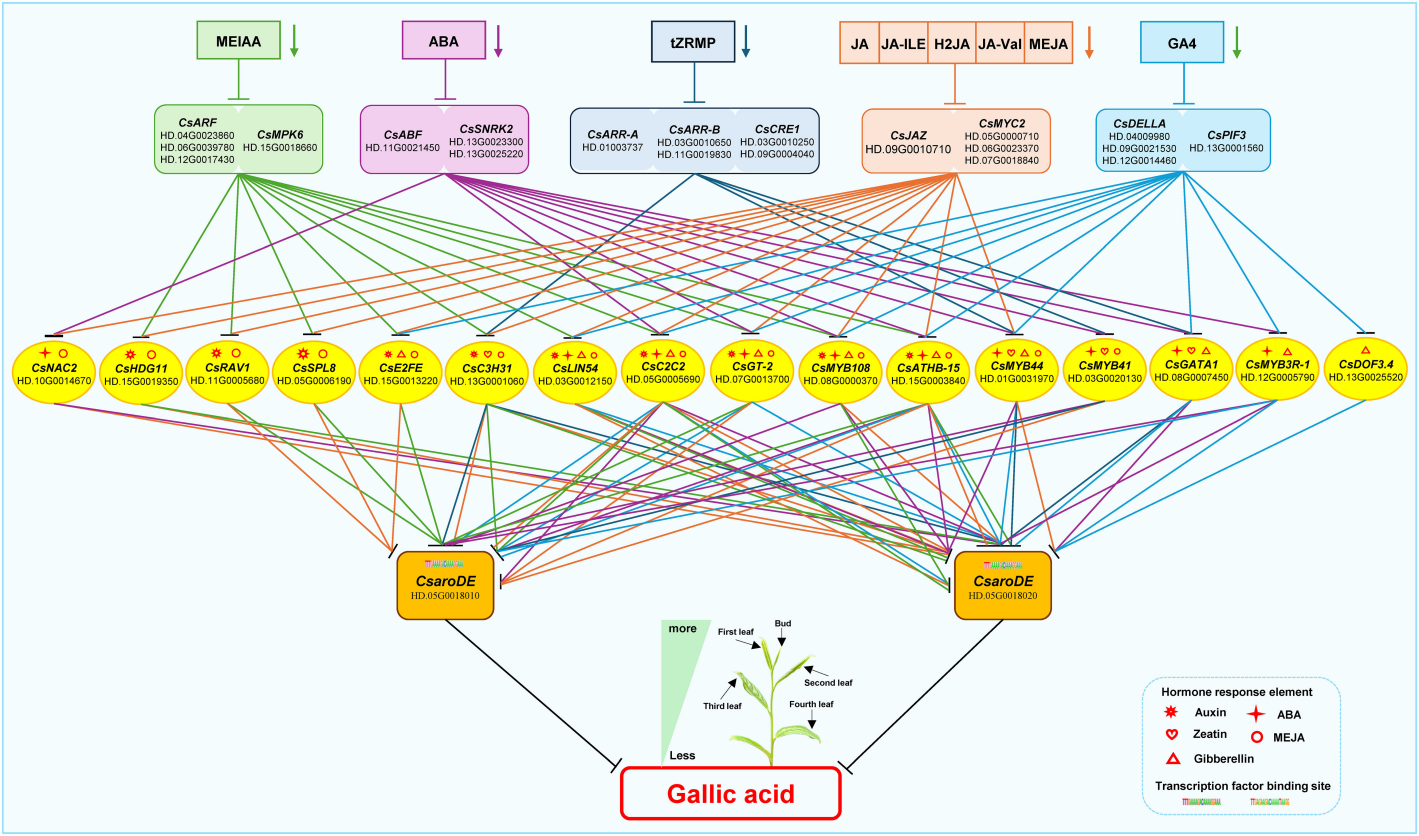
Proposed model map of endogenous hormones regulating GA biosynthesis during the development of buds and leaves in tea plant.

Based on the above results, the regulatory model of endogenous hormone regulating GA biosynthesis was constructed (**Figure 10**). During the development of tea shoots, endogenous hormones MEIAA, ABA, GA4, tZRMP, JA, JA-ILE, H2JA, JA-Val, and MEJA down-regulated the expression of *CsaroDE* via 16 transduction factors mediated by their signal transduction factors *CsARF, CsMPK6, CsABF, CsSNRK2, CsDELLA, CsPIF3, CsARR-A, CsARR-B, CsCRE1, CsJAZ*, and *CsMYC2*, respectively, and affect the biosynthesis of GA. Specifically, MEIAA down-regulated the expression of two *CsaroDE* via 10 transcription factors such as *CsHDG11*, *CsRAV1*, and *CsSPL8*, mediated by their signal transduction factors *CsARF* and *CsMPK6*, and inhibited the biosynthesis of GA. ABA down-regulated the expression of two *CsaroDE* via 9 transcription factors such as *CsNAC2*, *CsC2C2*, and *CsGT-2*, mediated by their signal transduction factors *CsABF* and *CsSNRK2*, and inhibited the biosynthesis of GA. GA4 down-regulated the expression of two *CsaroDE* and inhibited the biosynthesis of GA by 10 transcription factors such as *CsE2FE*, *CsLIN54* and *CsC2C2* mediated by signal transduction factors *CsDELLA* and *CsPIF3*. JA, JA-ILE, H2JA, JA-Val and MEJA down-regulated the expression of two *CsaroDE* and inhibited the biosynthesis of GA through 13 transcription factors such as *CsNAC2*, *CsHDG11* and *CsRAV1* mediated by signal transduction factors *CsJAZ* and *CsMYC2*.

#### 3.6.2 Endogenous jasmonic acid and abscisic acid jointly regulate the biosynthesis of GA

**Figure 11:**
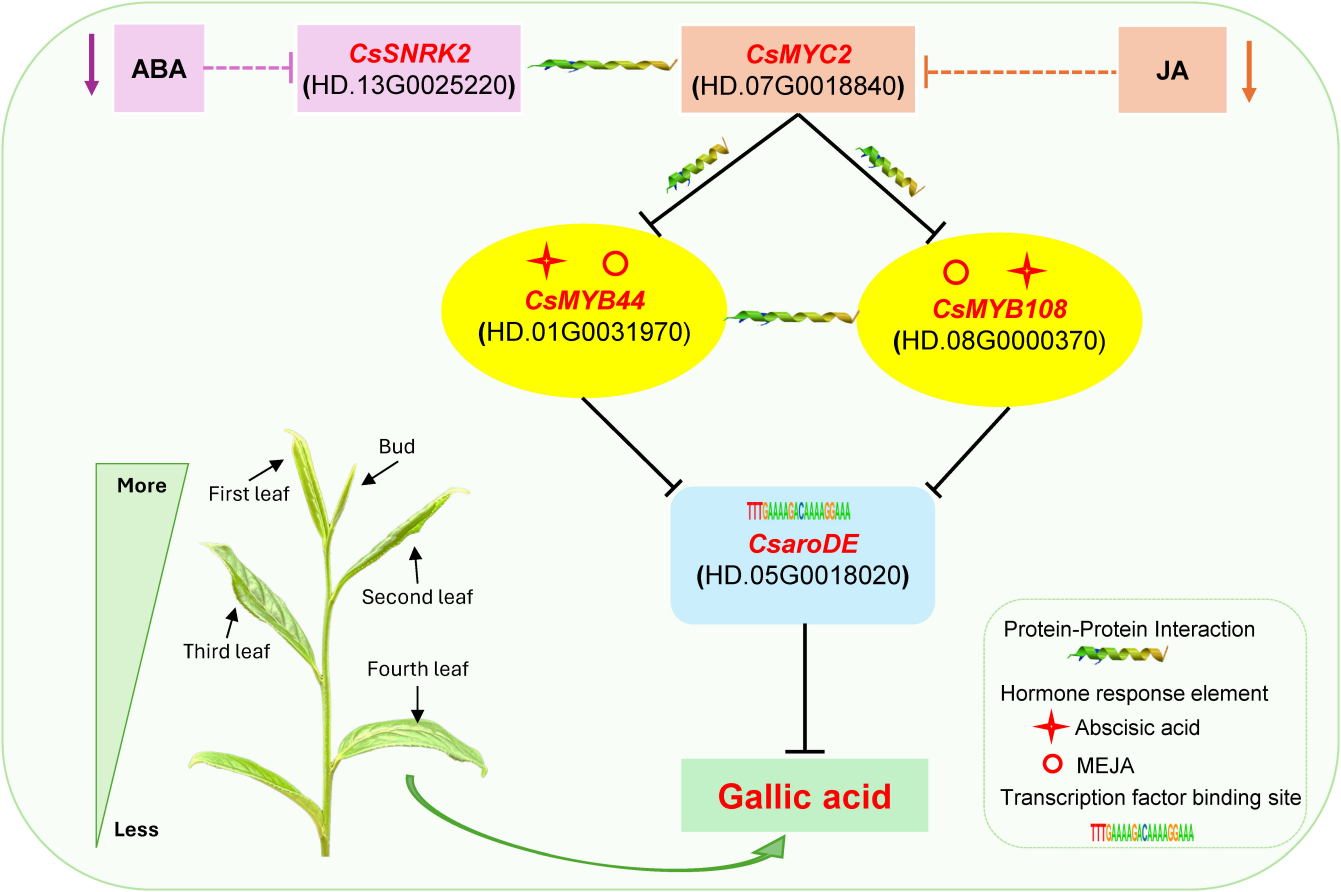
Potential mechanisms of endogenous JA and ABA co-regulation of GA biosynthesis.

Based on the results of endogenous hormone content analysis, it was hypothesized that endogenous JA and ABA might be the main endogenous hormones affecting the accumulation of GA content differences during the development of buds and leaves of tea shoots. Notably, among the 16 transcription factors that may be involved in the regulation of GA biosynthesis, the promoter regions of *CsLIN54*, *CsC2C2*, *CsGT-2*, *CsMYB108* and *CsATHB-15* contained auxin, abscisic acid, gibberellin and jasmonic acid response elements; the promoter region of *CsMYB44* contained abscisic acid, cytokinin, gibberellin and jasmonic acid response elements; the promoter region of *CsMYB41* contained abscisic acid, cytokinin and jasmonic acid response elements(**Supplementary Table 3**). It is speculated that these transcription factors may be capable of responding to two or more types of endogenous hormone signals, which in turn affect the biosynthesis of GA. According to the results of transcription factor binding site prediction and correlation analysis (**Figure 9**), five transcription factors, *CsMYB44*, *CsMYB108*, *CsC2C2*, *CsLIN54*, *CsATHB-15* all had binding sites with *CsaroDE* (HD.05G0018020), and their expression was significantly positively correlated with that of *CsaroDE*(HD.05G0018020); meanwhile, these five transcription factors showed significant positive correlation with four JA signaling factors and three ABA signaling factors. Protein-protein interaction (PPI) network analysis of these 10 genes using the String database (https://string-db.org/) showed that there was a protein interaction between *CsSNRK2* and *CsMYC2*, and there was a protein interaction between *CsMYC2* and *CsMYB44* and *CsMYB108* (**Supplementary Figure 1**). Accordingly, we hypothesized that *CsMYC2* may be a key cross-interacting site for integrating endogenous abscisic acid and jasmonic acid signaling, and *CsMYB44* and *CsMYB108* are key regulatory sites for regulating the expression of structural genes for GA biosynthesis in response to abscisic acid and jasmonic acid signaling. Based on these results, we constructed a map of the potential mechanism by which endogenous ABA and JA co-regulate GA synthesis during the development of buds and leaves of tea shoots. Specifically, the reduction of the content of endogenous ABA and JA inhibited the expression of *CsSNRK2*(HD.13G0025220) and *CsMYC2*(HD.07G0018840), and these two plant hormone signaling factors jointly downregulated the expression of *CsMYB44*(HD.01G0031970) and *CsMYB108*(HD.08G0000370) by forming a protein-protein interaction relationship, which in turn downregulated the expression of *CsaroDE*(HD.05G0018020), ultimately inhibiting the biosynthesis of GA (**Figure 11**).

## 4 Discussion

### 4.1 The decrease of D-erythrosyl-4-phosphate content and the increase of shikimic acid content affected the accumulation of GA content during the development of tea shoots

Gallic acid is synthesized from 3-dehydroshikimic acid, an intermediate product of the shikimic acid pathway, in a 2-step reaction in the presence of related enzymes (Kambourakis and Frost, 2000). Phosphoenolpyruvate (PEP) produced by the glycolysis (Embden-Meyerhof-Parnas, EMP) pathway and erythrose-4-phosphate (E-4P) produced by the pentose phosphate pathway (pentose phosphate pathway, PPP) are catalyzed by 3-deoxy-D-arabinoheptulose-7-phosphate synthase (DAHPS) to produce 3-deoxy-arabinoheptulose-7-phosphate (DAHP), which is then catalyzed by 3-dehydroquinic acid synthase (DHQS) to produce 3-dehydroquinic acid (DHQ). The intermediate product 3-dehydroshikimic acid (DHS) was further catalyzed by 3-dehydroquinic acid dehydratase/shikimate dehydrogenase (DHQ-SDH). Some of them were further dehydrogenated by shikimate dehydrogenase (SDH) to produce shikimic acid, and the other part was enolized to produce GA (Gross, 1999; Werner et al., 1999). The results of this study showed that the content of GA decreased gradually from Bud to L4, which was consistent with the results of previous studies (Zhou et al., 2020). The K-means clustering analysis of 53 plant hormone components and GA content demonstrated that the contents of 9 endogenous hormone components such as abscisic acid (ABA), auxin (MEIAA), cytokinin (tZRMP), gibberellin (GA4) and jasmonic acid (JA, JA-ILE, H2JA, JA-Val, MEJA) were significantly positively correlated with GA content, and the change pattern of these hormone contents was consistent with that of GA content. KEGG enrichment analysis of DAMs displayed that the DAMs in buds and leaves at different developmental stages were significantly enriched in shikimic acid pathway and plant hormone signal transduction pathway, demonstrating that the differential accumulation of GA in buds and leaves at different developmental stages of tea shoots may be related to the change of endogenous hormone content. Moreover, 2 DEGs related to GA biosynthesis, D-erythrose-4-phosphate and shikimic acid, were further identified from the DAMs. The content of D-erythrose-4-phosphate gradually decreased with the development of tea shoots and was positively correlated with GA content, the content of shikimic acid gradually increased with the development of tea shoots and was significantly negatively correlated with GA content, suggesting that the decrease in the content of D-erythrose-4-phosphate, the upstream substrate of the shikimic acid pathway, and the increase in the content of shikimic acid, a competitive metabolite of GA, affected the accumulation of GA content during the development of tea shoots.

### 4.2 Endogenous hormones regulate the expression of transcription factors through signal transduction factors, down-regulate the expression of structural genes of GA biosynthesis, and inhibit the biosynthesis of GA during the development of tea shoots

Plant hormones are involved in plant growth and development, as well as mediating plant responses to biotic and abiotic stresses as key endogenous factors (el Sabagh et al., 2022). Studies have found that plant hormones can control the expression of structural genes related to flavonoid synthesis by regulating the expression of transcription factors (Naik et al., 2022; He et al., 2023). Treatment of sweet cherry fruit with MEJA can increase the expression level of *PacMYBA* gene and up-regulate the transcription levels of *PacDFR*, *PacANS* and *PacUFGT*, thereby promoting the accumulation of anthocyanin in fruit (Shen et al., 2014). In this study, WGCNA analysis showed that *CsaroDE* was a key gene regulating GA biosynthesis during the development of tea shoots. Sixteen transcription factors had binding sites with *CsaroDE*, and their expression levels were highly correlated. The results of cis-acting element analysis showed that the promoter regions of 16 transcription factors contained multiple hormone response elements and were significantly positively correlated with 23 plant hormone signal transduction factors. The results of correlation analysis showed that these 23 plant hormone signal transduction factors were significantly correlated with the contents of 9 endogenous hormone components. Based on these results, we speculate that these 23 endogenous hormone signal transduction factors can respond to the changes of auxin, cytokinin, abscisic acid, gibberellin and jasmonic acid content during the development of tea shoots, and downregulate the expression of 16 transcription factors such as *CsMYB44*, *CsMYB108*, *CsC2C2*, thus downregulated the expression of *CsaroDE*, thereby inhibiting the biosynthesis of GA. This result is consistent with the findings of several previous studies. In apple, MeJA can promote anthocyanin accumulation by inducing the overexpression of *MdMYB9* or *MdMYB11* (An et al., 2015). IAA mediated *CsWRKY* through *CsIAA* and *CsSAUR* to promote the expression of *CsCHS* and *CsANS* and increase the content of EGCG in tea plant. *CsC2H2*, *CsSBP* and *CsWRKY* mediated by IPR and IP inhibited the synthesis of EGCG through *CsARR-A* and *CsARR-B* (Zhu et al., 2023).

### 4.3 JA and ABA co-regulate GA biosynthesis during the development of buds and leaves of tea shoots

Interactions among different plant hormones are crucial for the growth of higher plants, and the crosstalk regulatory network of plant hormones has received increasing attention because of its unique function in plants (Aerts et al., 2021). In crosstalk of phytohormones, one hormone regulates physiological processes in higher plants through interactions with other phytohormones (Murphy, 2015). Plants in nature and agriculture have evolved a complex and flexible environmental network of phytohormone-directed signals in order to survive under different conditions (Berens et al., 2017). Salicylic acid, jasmonic acid, ethylene, and abscisic acid interact to regulate plant defence responses (Yang et al., 2015; Li et al., 2019). Previous studies have shown that JA treatment significantly promotes GA content in germinated buckwheat (*Fagopyrum esculentum* Moench) (Park et al., 2019); Gallic acid content in *Populus deltoides* leaves treated with MeJA comes to a peak at 24 h in turnip (*Brassica rapa* ssp. *rapa*) (An et al., 2006); gallic acid was present in JA and ABA-treated plants but was not exhibited in the control plants (Thiruvengadam et al., 2016); ABA treatment could significantly promote the increase of GA content in strawberry leaves under different salt stress conditions (Jamalian et al., 2013); ABA induced high levels of gallic acid in the callus of sugarcane exposed at the maturation process both, alone and in combination with JA (Nieves et al., 2001). These findings shown that both JA and ABA can promote the increase of GA content, and there may be an interaction between the two hormones in inducing the increase of GA content. In this study, the results of OPLS-DA analysis of endogenous hormone content and GA content showed that JA and ABA may be the key hormones affecting GA biosynthesis during the development of tea shoots. Further analysis by WGCNA revealed that among the 16 transcription factors that may be involved in GA biosynthesis, the promoter regions of five transcription factors including *CsMYB44*, *CsMYB108*, and *CsC2C2* contained both abscisic acid and jasmonic acid response elements, and these transcription factors were significantly positively correlated with four JA signal transduction factors and three ABA signal transduction factors. PPI analysis showed that there was a protein interaction between *CsMYC2*(HD.07G0018840) and *CsSNRK2*(HD.13G0025220), and *CsMYC2* (HD.07G0018840) has protein interactions with *CsMYB44*(HD.01G0031970) and *CsMYB108*(HD.08G0000370), respectively. Based on these results, we concluded that during the development of tea shoots, the decrease of endogenous JA and ABA content caused the downregulation of *CsMYC2*(HD.07G0018840) and *CsSNRK2*(HD.13G0025220), and the two plant hormone signal transduction factors down-regulated the expression of *CsMYB44* and *CsMYB108* (HD.08G0000370) through protein-protein interaction, and then down-regulated the expression of *CsaroDE*(HD.05G0018020), thus jointly inhibited the biosynthesis of GA. We speculate that *CsMYC2* may be a key interaction site for integrating JA and ABA signaling pathways to regulate GA biosynthesis. Previous studies have found similar results that jasmonic acid, salicylic acid, abscisic acid, and ethylene form a crosstalk network through two key transcription factors, *MYC2* and *ORA59* (Liu and Timko, 2021; Yang et al., 2021). A recent study shows that gallic acid increased by 60% in *PgMyb308-lik*e-overexpressing *A. thaliana* lines, suggesting that the transcription factor MYB plays an important role in the regulation of GA biosynthesis (Dhakarey et al., 2022). Our study also found that *CsMYB44* and *CsMYB108* may be key regulatory sites for endogenous JA and ABA to jointly regulate GA biosynthesis, which is consistent with previous findings. The specific mechanism of the interaction between JA and ABA in regulating GA biosynthesis needs further study.

## 5 Conclusion

In summary, during the development of tea shoots from bud to the fourth leaf, the decrease of D-erythrosyl-4-phosphate content and the increase of shikimic acid content affected the accumulation of GA content. Jasmonic acid, abscisic acid, auxin, cytokinin and gibberellin may down-regulate the expression of two *CsaroDE* by 16 transcription factors such as *CsMYB44*, *CsMYB108* and *CsC2C2* through their signal transduction factors, thus inhibiting the biosynthesis of GA. Further analysis showed that *CsMYC2*(HD.07G0018840) interacted with *CsSNRK2*(HD.13G0025220), downregulated the expression of transcription factors *CsMYB44*(HD.01G0031970) and *CsMYB108*(HD.08G0000370), and inhibited the expression of *CsaroDE*(HD.05G0018020), thereby jointly regulating the biosynthesis of GA. Our findings are helpful to elucidate the potential mechanism of endogenous hormones regulating GA biosynthesis during the development of buds and leaves of tea shoots and provide a theoretical basis for the quality control of fresh tea leaves.

## Data Availability

The raw data of RNA-Seq underlying this study are openly available in the SAR database under project accession PRJNA1201555 (https://www.ncbi.nlm.nih.gov/sra/PRJNA1201555).
